# The change of cervical sagittal parameters plays an important role in clinical outcomes of cervical spondylotic myelopathy after multi-level anterior cervical discectomy and fusion

**DOI:** 10.1186/s13018-019-1504-3

**Published:** 2019-12-11

**Authors:** Xi-Wen Fan, Zhi-Wei Wang, Xian-Da Gao, Wen-Yuan Ding, Da-Long Yang

**Affiliations:** Department of Spinal Surgery, The Third Hospital of Hebei Medical University, 139 Ziqiang Road, Shijiazhuang, 050051 People’s Republic of China

**Keywords:** Risk factors, Clinical outcomes, Multi-level cervical spondylotic myelopathy, Anterior cervical discectomy and fusion, Cervical sagittal parameters

## Abstract

**Background:**

Cervical sagittal parameters were closely related with clinical outcomes after multi-level ACDF. Our purpose was to evaluate the clinical outcomes and cervical sagittal parameters in patients with MCSM after ACDF and to identify the risk factors of poor clinical outcomes.

**Material and methods:**

ACDF was performed in 89 patients with MCSM. Based on average JOA recovery rate, patients were divided good-outcome group (group GO) and poor-outcome group (group PO). The cervical sagittal parameters including Cobb angle, SVA, T1S, cranial tilt and cervical tilt were measured. Multivariate logistic regression was used to identify risk factors.

**Results:**

Fifty-four patients (60.67%) were divided into group GO, while 35 patients (39.33%) were divided into group PO. Cobb angle, SVA and T1S was corrected from preoperative average 11.80° ± 9.63°, 23.69 mm ± 11.69 mm and 24.43° ± 11.78° to postoperative average 15.08° ± 9.05°, 18.79 mm ± 10.78 mm and 26.92° ± 11.94° respectively (*p* < 0.001). △Cobb angle (*p* = 0.008) and △SVA (*p* = 0.009) showed significantly statistical differences between two groups. Longer symptom duration, lower preoperative JOA score, smaller △Cobb angle and larger △SVA were identified as risk factors of poor clinical outcomes.

**Conclusion:**

Multi-level ACDF is an effective surgical method to treat patients with MCSM. However, long duration of preoperative symptoms, lower preoperative JOA score, smaller △Cobb angle and larger △SVA are risk factors for poor outcomes in patients with MCSM after ACDF. Sagittal parameters should be paid attention to in surgery.

## Introduction

Cervical spondylotic myelopathy (CSM) is caused by spinal cord compression as a result of multiple pathological changes such as disc herniation, degeneration and/or osteophyte formation at the posterior margin of the vertebral body [1]. Multi-level cervical spondylotic myelopathy (MCSM) refers to spinal cord compression more than 3 levels in CSM. MCSM leads to varying degrees of symptoms including spastic tetraparesis and sensory dysfunction. In principle, the procedure should be performed in time to relieve the compression and protect spinal function [2]. For 1–2 segments CSM, anterior cervical discectomy and fusion (ACDF) is the most common cervical fusion surgery [3, 4]. However, the choice of surgical methods for MCSM is still controversial, which mainly focuses on anterior procedure or posterior procedure [5, 6]. Posterior procedure was safe and easy to perform; however, indirect decompression would not remove the herniated disc, which led to uncertain long-term outcomes [7, 8]. High incidence of complications, axial symptoms and how to restore and maintain cervical physiological curvature were also the problems before making the decision to perform a posterior cervical surgery [7, 8]. Anterior procedure removed the compressed disc directly and was proved better clinical outcomes blood loss, shorter operation time and better cervical curvature [5, 6].

As an anterior procedure surgery, multi-level ACDF was widely used in treating MCSM [9]. Although satisfactory clinical prognosis was achieved as a whole, some of patients were not benefitted from the surgery and suffered from persistent neurological symptoms or even worse. Cervical sagittal parameters, including cervical lordosis (CL) (that is Cobb angle in our study), C2-C7 sagittal vertical axis (SVA) and T1 slope (T1S) were proved to be related with clinical outcomes after ACDF in patients with CSM [10, 11]. Many previous studies had discussed different cervical sagittal parameters in healthy people or different cervical diseases [10, 12]; however, few studies have focused on the correlations between cervical sagittal parameters and clinical outcomes, especially in patients with MCSM after ACDF. So, the aim of the study was to evaluate the clinical outcomes and cervical sagittal parameters in patients with MCSM after ACDF and to identify the risk factors of poor clinical outcomes, which could help to make reasonable surgical program and achieve better clinical outcomes.

## Materials and methods

### Patients

All protocols of the study were approved by the Ethics Committee of the Third Hospital of Hebei Medical University and informed consent was obtained from all individual participants for using their imaging data and questionnaire scores.

From January 2010 to December 2015, 89 patients, including 40 men and 49 women, diagnosed as MCSM according to clinical manifestations and imaging scans underwent multi-level ACDF at the Department of Spinal Surgery, the Third Hospital of Hebei Medical University, were enrolled in this retrospective study. The inclusion criteria were the following: (1) MCSM required surgical treatment for equal to or more than three levels; (2) ineffective conservative treatment for more than 3 months or acute aggravated neurological deficit; (3) complete imaging and clinical date; (4) follow-up for at least 2 years. Exclusion criteria were the following: (1) history of operation involving with cervical spine; (2) combined with trauma, spinal tumours, spinal tuberculosis or infections; (3) ossification of posterior longitudinal ligament; (4) combined with severe osteoporosis; (5) combined with neurological diseases, such as vitamin B deficiency or motor neuron diseases. The average age of all patients at operation was 58.97 ± 5.79 years, range from 37 to 78 years. Three-level ACDF (C3-C6 in 34 cases and C4-C7 in 41 cases) was performed in 75 patients and four-level ACDF (C3-C7) in 14 patients. The patients were followed up for an average of 2.57 ± 0.78 years.

### Clinical and imaging assessment

Japanese Orthopaedic Association (JOA) scoring system (0–17 scores) was used for neurological function assessment before surgery and at last follow-up visit. Neurological function recovery rate was calculated on the basis of JOA scoring system: (postoperative score-preoperative score)/(17-preoperative score) × 100%. According to average JOA scores, patients were divided into two groups: good-outcome group (group GO, recovery rate greater than the average) and poor-outcome group (group PO, recovery rate lower than the average).

**Fig. 1:**
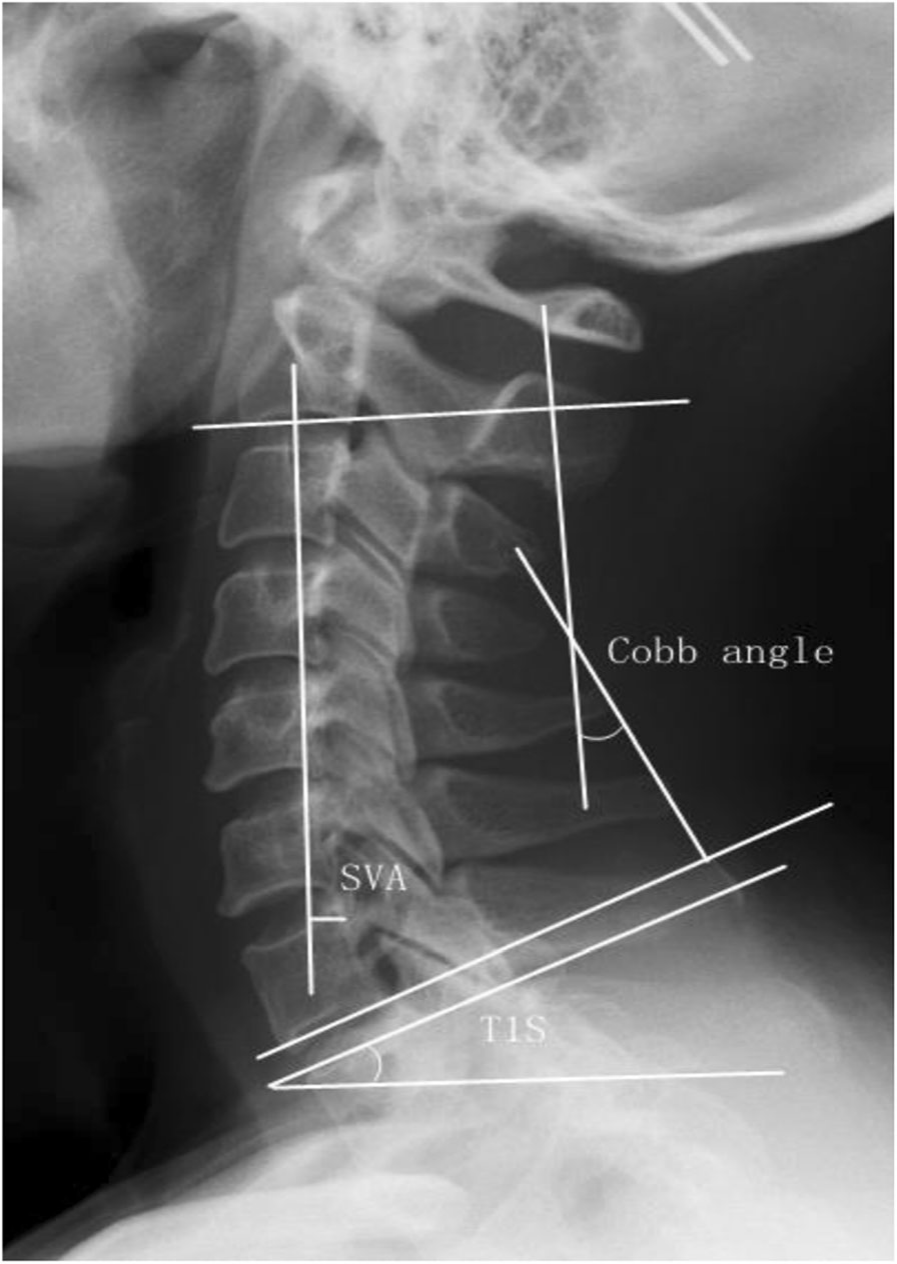
Measurement of cervical sagittal parameters. Cobb angle: angle between inferior endplate of C2 vertebra and inferior endplate of C7 vertebra; SVA: the horizontal distance between plumb line from C2 vertebra central point and posterosuperior corner of C7 vertebra; T1S: the angle between horizontal line and inferior endplate of T1 vertebra

**Fig. 2:**
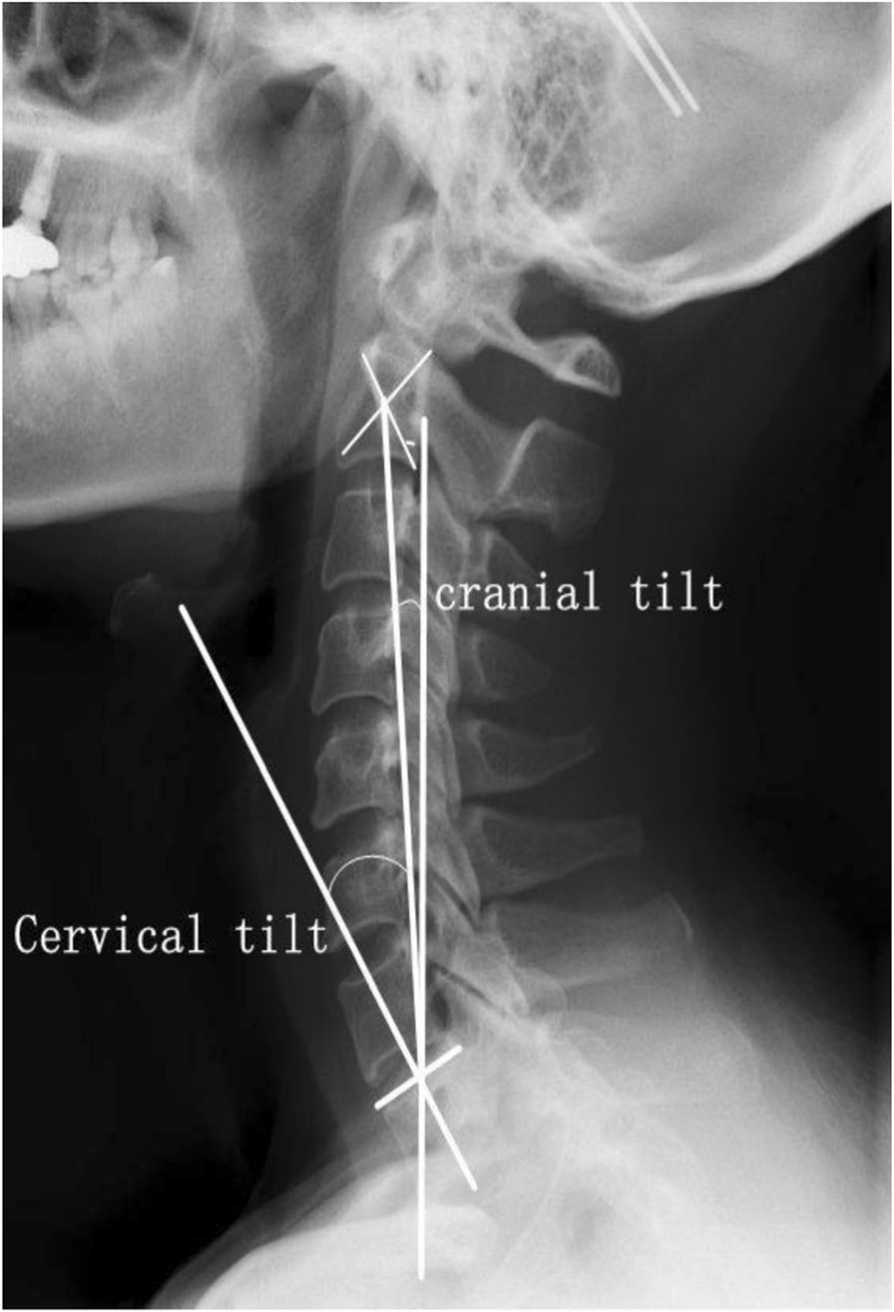
Measurement of cervical sagittal parameters. Cranial tilt: the angle between the line extending from the centre of the T1 endplate to the tip of the dens and the plumb line; cervical tilt: the angle between the line extending from the centre of the T1 endplate to the tip of the dens and the vertical line from the centre of the T1 endplate

Cervical magnetic resonance imaging (MRI), CT (Computed Tomography) and posterior-anterior and lateral X-rays were taken preoperatively to diagnose and evaluate the disease and make surgical planning. Cervical posterior-anterior and lateral X-rays were also taken postoperatively and at each follow-up visit. Cervical sagittal parameters were measured on lateral radiographs on synapse system (version 3.2.1; FUJIFILM MEDICAL SYSTEMS, U.S.A., INC, Stamford) with patients in a neutral position. The measurement methods of cervical sagittal parameters were as follows (Figs. 1 and 2): (1) Cobb angle: angle between inferior endplate of C2 vertebra and inferior endplate of C7 vertebra; (2) sagittal vertical axis (SVA): the horizontal distance between plumb line from C2 vertebra central point and posterosuperior corner of C7 vertebra; (3) T1 slope (T1S): the angle between horizontal line and inferior endplate of T1 vertebra; (4) cranial tilt: the angle between the line extending from the centre of the T1 endplate to the tip of the dens and the plumb line; (5) cervical tilt: the angle between the line extending from the centre of the T1 endplate to the tip of the dens and the vertical line from the centre of the T1 endplate. △Cobb angle, △SVA, △T1S, △cranial tilt and △cervical tilt were defined as the difference between last follow-up visit and preoperative measured values, respectively.

Patients’ age, gender, body mass index (BMI), duration of symptoms, follow-up period, preoperative JOA scores, number of operative segments, △Cobb angle, △SVA, △T1S, △cranial tilt and △cervical tilt were collected for potential risk factors of poor clinical outcomes in patients with MCSM after ACDF. Duration of symptoms was defined as the period from appearance of primary neurological clinical symptoms to surgery.

### Statistical analysis

Clinical and imaging date was evaluated by SPSS program (version 22.0; SPSS Inc., Chicago, IL, USA). *p* value < 0.05 was considered statistically significant. Quantitative dates were first tested its normality and homogeneity of variance and according to different situations, they were tested by Student’s *t* test or Mann–Whitney *U* test. Qualitative date was tested by chi-square test. The potential risk factors were tested by univariate analysis and if *p* < 0.05, the factor was selected into multivariate logistic model. Then, multivariate logistic regression analysis was used to identify the risk factors of poor clinical outcomes in patients with MCSM after ACDF with adjusted odds ratios (ORs) and 95% confidence intervals (CIs).

## Results

### Clinical outcomes

**Fig. 3:**
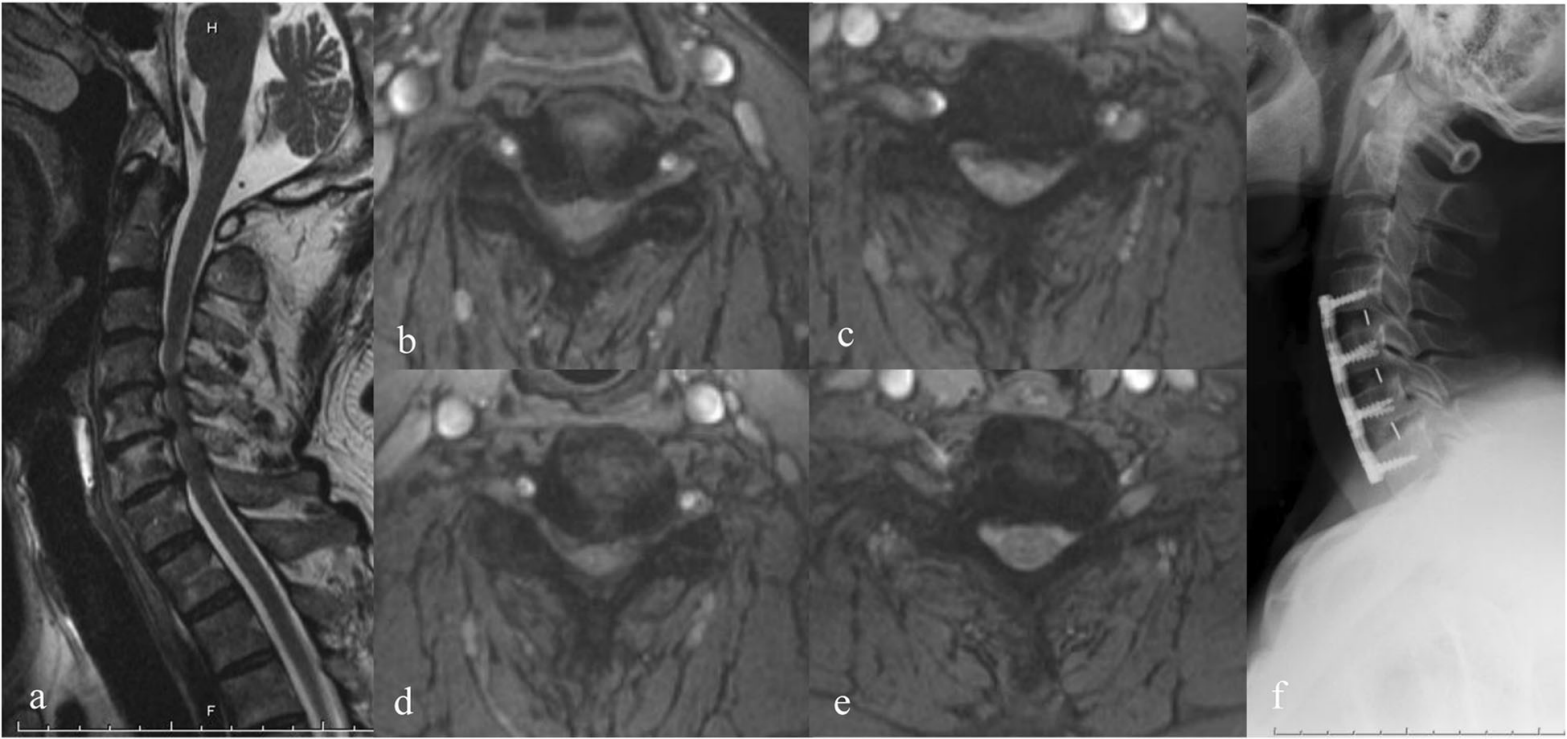
ACDF treatment of MCSM. **a** A 65-year-old male patient was diagnosed as MCSM with typical symptoms of superior motor neurons compression. T2-weighted sagittal MRI showed spinal cord compression in C4/5, C5/6, C6/7. **b**–**e** C4/5, C5/6, C5/6, C6/7 spinal cord compression on T2-weighted axial MRI. **f** Multi-level ACDF was performed to release the compression. Lateral X-ray of cervical spine was taken at 2-year follow-up visit and sagittal parameters were corrected appropriately

For all patients, the mean duration of symptoms was 14.64 ± 8.02 months. All the operations were completed successfully (Fig. 3). JOA score at last follow-up visit (13.16 ± 2.74) was significantly higher than preoperative scores (7.73 ± 2.84) (*p* < 0.001). The average recovery rate was 61.13% ± 21.48%. Fifty-four patients (60.67%) whose recovery rate higher than the average were divided into group GO and their average recovery rate was 75.00% ± 9.52%, while 35 patients (39.33%) whose recovery rate lower than the average were divided into group PO and their average recovery rate was 39.73% ± 16.62%. There was significantly statistical difference in recovery rate between two groups (*p* < 0.001).

The clinical dates between group GO and group PO were showed in Table 1. There were no significantly statistical differences found in age (*p* = 0.114), sex (*p* = 0.450), BMI (*p* = 0.582), follow-up time (*p* = 0.159) and operative segment (*p* = 0.768) between two groups. The JOA scores were significantly improved at last follow-up visit comparing with the preoperative scores in both groups (*p* < 0.001). No matter preoperative JOA scores (*p* = 0.014) or last follow-up JOA scores (*p* < 0.001), group GO got significantly higher scores than group PO. In addition, patients in group PO complained of syndromes for a significantly longer period before surgery than patients in group GO (*p* < 0.001).

**Table 1:** Comparison of patient characteristics between group GO and group PO

	Total (*n* = 89)	Group GO (*n* = 54)	Group PO (*n* = 35)	t/z	*p*
Age (year)	58.97 ± 5.79	58.19 ± 5.68	60.17 ± 5.82	1.596	0.114
Sex (male/female)	40/49	26/28	14/21	0.570	0.450
BMI	25.74 ± 4.00	25.47 ± 3.53	26.17 ± 4.66	0.550	0.582
Duration of symptoms (months)	14.64 ± 8.02	11.41 ± 5.42	19.63 ± 8.85	4.618	< 0.001
Follow-up time (years)	2.57 ± 0.78	2.45 ± 0.64	2.76 ± 0.94	1.410	0.159
JOA score					
Preoperative	7.73 ± 2.84	8.39 ± 2.60	6.17 ± 2.94	2.468	0.014
Last follow-up	13.16 ± 2.74	14.74 ± 1.12	10.71 ± 2.72	7.205	< 0.001
t/z	8.206	6.414	5.144		
*p*	< 0.001	< 0.001	< 0.001		
Recovery rate	61.13% ± 21.48%	75.00% ± 9.52%	39.73% ± 16.62%	7.948	< 0.001
Operative segments				0.087	0.768
Three	75	46	29		
Four	14	8	6		

### Cervical sagittal parameters

For all patients, Cobb angle was corrected from preoperative average 11.80° ± 9.63° to postoperative average 15.08 ± 9.05° (*p* < 0. 001) and the average △Cobb angle was 3.28° ± 3.88°. The average last follow-up SVA (18.79 mm ± 10.78 mm) was significantly lower than the preoperative (23.69 mm ± 11.69 mm) (*p* < 0.001) and the average △SVA was − 4.90 mm ± 6.30 mm. Meanwhile, significantly significant difference was also found between preoperative T1S (24.43° ± 11.78°) and last follow-up T1S (26.92° ± 11.94°) (*p* < 0.001) and the average △T1S was 2.49° ± 4.19°. Significantly statistical difference (*p* < 0.001) was found between preoperative (5.16° ± 6.47°) and last follow-up cranial tilt (7.52° ± 6.27°) and the average △cranial tilt was 2.36° ± 2.25°.There was no significantly statistical difference (*p* = 0.132) between preoperative (17.71° ± 6.28°) and last follow-up cervical tilt (17.46° ± 6.54°) and the average △cervical tilt was − 0.25° ± 1.53°.

The comparison of cervical sagittal parameters between group GO and group PO is shown in Table 2. There was no significantly statistical difference in preoperative Cobb angle (*p* = 0.467), preoperative SVA (*p* = 0.868) and preoperative T1S (*p* = 0.740) between two groups. However, last follow-up Cobb angle in group GO was greater than that in group PO (*p* = 0.025) and significantly statistical difference was also found when comparing △Cobb angle (*p* = 0.008). Last follow-up SVA showed the opposite result than the value in group PO was greater than that in group GO (*p* = 0.030). △SVA in group PO was also greater than that in group GO (*p* = 0.009). There was no significantly statistical difference in both last follow-up T1S (*p* = 0.814) and △T1S (*p* = 0. 826) between two groups. No significantly statistical difference was found in preoperative cranial tilt (*p* = 0.740), last follow-up cranial tilt (*p* = 0.653), △cranial tilt (*p* = 0.952), preoperative cervical tilt (*p* = 0.590), last follow-up cervical tilt (*p* = 0.585) and △cervical tilt (*p* = 0.946) between two groups.

**Table 2:** Comparison of cervical sagittal parameters between group GO and group PO

	Total (*n* = 89)	Group GO (*n* = 54)	Group PO (*n* = 35)	t/z	*p*
Cobb angle (°)					
Preoperative	11.80 ± 9.63	12.35 ± 9.75	10.94 ± 9.51	0.727	0.467
Last follow-up	15.08 ± 9.05	16.48 ± 9.18	12.91 ± 8.52	2.246	0.025
t/z	6.191	5.366	3.120		
*p*	< 0.001	< 0.001	0.002		
△Cobb angle (°)	3.28 ± 3.88	4.13 ± 4.11	1.97 ± 3.11	2.633	0.008
SVA (mm)					
Preoperative	23.69 ± 11.69	23.52 ± 10.89	23.94 ± 12.98	0.166	0.868
Last follow-up	18.79 ± 10.78	16.76 ± 9.96	21.91 ± 11.39	2.168	0.030
t/z	6.484	6.385	2.338		
*p*	< 0.001	< 0.001	0.019		
△SVA (mm)	− 4.90 ± 6.30	− 6.76 ± 5.41	− 2.03 ± 6.57	2.629	0.009
T1S (°)					
Preoperative	23.43 ± 11.78	24.69 ± 11.29	24.03 ± 12.65	0.332	0.740
Last follow-up	26.92 ± 11.94	27.31 ± 11.06	26.31 ± 13.32	0.235	0.814
t/z	4.712	4.627	2.759		
*p*	< 0.001	< 0.001	0.006		
△T1S (°)	2.49 ± 4.19	2.63 ± 4.18	2.29 ± 4.27	0.219	0.826
Cranial tilt					
Preoperative	5.16 ± 6.47	5.31 ± 6.08	4.91 ± 7.11	0.332	0.740
Last follow-up	7.52 ± 6.27	7.80 ± 6.12	7.09 ± 6.56	0.450	0.653
t/z	6.651	5.850	3.471		
*P*	< 0.001	< 0.001	0.001		
△Cranial tilt	2.36 ± 2.25	2.48 ± 1.88	2.17 ± 2.74	0.060	0.952
Cervical tilt					
Preoperative	17.71 ± 6.28	18.06 ± 6.12	17.17 ± 6.57	0.539	0.590
Last follow-up	17.46 ± 6.54	17.87 ± 6.56	16.83 ± 6.55	0.545	0.585
t/z	1.522	0.896	1.291		
*p*	0.132	0.374	0.205		
△Cervical tilt	− 0.25 ± 1.53	− 0.19 ± 1.52	− 0.34 ± 1.57	0.067	0.946

### Risk factors for poor outcome of ACDF for MCSM

Symptom duration (*p* < 0.001), preoperative JOA score (*p* = 0.009), △Cobb angle (*p* = 0.013) and △SVA (*p* = 0.001) showed significantly statistical difference in univariate analysis and the four factors were selected into multivariate logistic model (Table 3). In multivariate logistic regression analysis, longer symptom duration (OR = 1.248, 95% CI = 1.113–1.398, *p* < 0.001), lower preoperative JOA score (OR = 0.593, 95% CI = 0.427–0.824, *p* = 0.002), smaller △Cobb angle (OR = 0.793, 95% CI = 0.667–0.944, *p* = 0.009) and larger △SVA (OR = 1.227, 95% CI = 1.060–1.421, *p* = 0.006) were identified as four risk factors of poor clinical outcomes in patients with MCSM after ACDF (Table 3).

**Table 3:** Multivariate logistic regression analysis of poor clinical outcomes of ACDF for MCSM

	*B*	Se	Wald	*p*	OR	95% CI
Duration of symptoms	0.221	0.058	14.477	< 0.001	1.248	1.113–1.398
Preoperative JOA score	0.522	0.167	9.736	0.002	0.593	0.427–0.824
△Cobb angle	0.232	0.089	6.825	0.009	0.793	0.667–0.944
△SVA	0.205	0.075	7.490	0.006	1.227	1.060–1.421

## Discussion

Cervical spondylotic myelopathy (CSM) is one of the most common diseases in orthopaedics and is one of the most harmful diseases, mostly in the elderly [1]. It has the characteristics of concealment and intermittence and when the symptoms are serious, patients may lose their normal life or working ability [13]. Currently, different methods of anterior surgery is commonly used to treat MCSM, including anterior cervical discectomy and fusion (ACDF), anterior cervical corpectomy and fusion (ACCF) and anterior cervical hybrid decompression and fusion (ACHDF), and the similar clinical outcomes was showed among them [14, 15]. However, in treatment of MCSM by three types of anterior surgery, ACDF acquired the lowest incidence rate of complications as 15.53%, while the incidence rate in ACCF and ACHDF were 26.44% and 22.92%, respectively [16]. Although decompression of ACCF and ACHDF was more adequate, the two types of anterior surgery caused greater damage to the anterior and central column of cervical spine and have trouble restoring the cervical physiological curvature [17, 18]. Moreover, the extension of the bone graft distance in ACCF and ACHDF caused greater risk of complications such as bone graft nonfusion, pseudoarthrosis formation, loose internal fixation and implant settlement [16, 18]. In addition, with accurate decompression, ACDF had advantage of shorter operation time, less blood loss and less trauma to patients compared with ACCF [17, 18].

The clinical outcomes of ACDF were limited by a variety of factors involving with preoperative condition and postoperative complications. Pumberger et al. [19] found that the postoperative outcomes were associated with duration of symptoms, age, BMI and preoperative MRI spinal cord signal changes. Several studies indicated that cervical JOA scores and age were predictive of outcome after decompressive surgery for CSM [20, 21]. In our study, ACDF surgery was performed in 89 MCSM patients with an average follow-up visit of 2.57 years and the results showed neurological function was significantly improved at the last follow-up visit. However, there were still some patients with poor postoperative neurological recovery and recovery rate the patients in group PO was only 39.73% ± 16.62%. Multivariate logistic analysis showed long duration of symptoms, lower preoperative JOA score, smaller △Cobb angle and larger △SVA. Preoperative neurological status was closely related to postoperative neurological recovery. Sever compression in cervical spinal cord caused irreversible neurological deficiency, so the neurological function would not recover, even after adequate decompression.

Cervical sagittal parameters had been proved to be important in clinical recovery of patients with CMS after cervical surgery and preoperative cervical sagittal parameters had been proved to be predictors for clinical outcomes [22–24]. Cervical sagittal parameters usually consisted of Cobb angle, SVA and T1S, and they played different roles during the whole treatment of CSM [10, 24–28]. Cobb angle was used to describe cervical curvature and was easily affected by the disc degeneration which was one of the manifestations in patients with MCSM [10]. SVA was a cervical sagittal parameter to evaluate cervical sagittal balance and value of normal asymptomatic volunteers was maintained within a narrow range of 20 mm and cervical sagittal imbalance was defined as the value greater than 40 mm [26, 27, 29]. T1S was used to describe the relationship between the T1 vertebra and cervical lordosis and it significantly influenced by flexion and extension of the neck [28]. It was reported that Cobb angle and T1S were both significantly increased after double-segment ACDF surgery [30, 31]. However, the change of SVA was still controversial. In the study of Huang Y et al. [31], there was no significantly statistical difference in SVA before and after ACHDF of MCSM, while Gillis et al. [32] reported that the postoperative SVA was significantly lower than the preoperative after anterior surgery. The different results attributed to the different inclusion criteria, surgery methods and follow-up time. The three cervical sagittal parameters were not independent from each other. Yuan et al. [32] found there was negative correlation between SVA and Cobb angle, and cervical lordosis was the only predictor of SVA and cervical sagittal parameters was associated with symptoms. In our study, Cobb angle and T1S were significantly increased at the last follow-up visit after ACDF in patients with MCSM, while SVA was significantly decreased at the last follow-up visit, and △Cobb angle and △SVA were closely related to the postoperative clinical outcomes.

The similar result showed in the study of Basques et al. [9] that ACDF surgery could partially restore cervical physiological lordosis of patients with multi-segmental cervical spondylosis. In ACDF surgery, anterior and middle column of cervical spine was distracted by cage with autogenous bone in collapsed intervertebral space and in this way, the Cobb angle was partially corrected. The similar results found in the study of Gum et al. [33] that maintenance or reconstruction of cervical lordosis was conducive to achieve good outcome after ACDF surgery. SVA was another cervical sagittal parameter closely related to clinical outcomes [30]. Lee et al. [12] found that SVA was an effective predictor of quality of life. Our study showed patients with increased Cobb angle and decreased SVA after surgery were more likely to achieve better clinical outcomes. Smaller △Cobb angle and larger △SVA were risk factors for poor postoperative recovery in patients with MCSM; however, TIS was independent from neurological function recovery. How to correct cervical sagittal parameters should be considered when making surgical plan to treat MCSM for better clinical outcomes.

## Conclusions

Multi-level ACDF is an effective surgical method to treat patients with MCSM. Cervical sagittal parameters were changed after multi-level ACDF with larger Cobb angle, smaller SVA and greater T1S. However, long duration of preoperative symptoms, lower preoperative JOA score, smaller △Cobb angle and larger △SVA is risk factors for poor outcomes in patients with MCSM after ACDF. Sagittal parameters should be paid attention to in design of surgical plan for better clinical outcomes.

## Data Availability

The datasets generated and analysed during the current study are available from the corresponding author on reasonable request.

## Abbreviations

ACCF: Anterior cervical corpectomy and fusion
ACDF: Anterior cervical discectomy and fusion
ACHDF: Anterior cervical hybrid decompression and fusion
BMI: Body mass index
CL: Cervical lordosis
CSM: Cervical spondylotic myelopathy
JOA: Japanese Orthopaedic Association
MCSM: Multi-level cervical spondylotic myelopathy
SVA: C2-C7 sagittal vertical axis
T1S: T1 slope
